# Blood biomarkers for vascular cognitive impairment based on neuronal function: a systematic review and meta-analysis

**DOI:** 10.3389/fneur.2025.1496711

**Published:** 2025-02-07

**Authors:** Weiquan Huang, Libin Liao, Qian Liu, Rongchao Ma, Xuan He, Xiaoqiong Du, Dujuan Sha

**Affiliations:** ^1^Department of General Practice, Nanjing Drum Tower Hospital Clinical College of Nanjing Medical University, Nanjing, Jiangsu, China; ^2^Department of General Practice, Nanjing Drum Tower Hospital Clinical College of Xuzhou Medical University, Nanjing, Jiangsu, China; ^3^Department of General Practice, Nanjing Drum Tower Hospital, Affiliated Hospital of Medical School, Nanjing University, Nanjing, China; ^4^State Key Laboratory of Pharmaceutical Biotechnology, Institute of Functional Biomolecules, Nanjing University, Nanjing, China

**Keywords:** vascular cognitive impairment, biomarkers, blood, neuronal function, systematic review

## Abstract

Vascular cognitive impairment (VCI) is increasingly recognized as the second most prevalent cause of dementia, primarily attributed to vascular risk factors and cerebrovascular disease. Numerous studies suggest that blood biomarkers may play a crucial role in the detection and prognosis of VCI. This study conducted a meta-analysis to evaluate the potential of various blood biomarkers associated with neuronal function as indicators of VCI. We searched four major databases—PubMed, Embase, Web of Science, and the Cochrane Library—up to December 31, 2023, for research on blood biomarkers for VCI. Of the 4,043 studies identified, 30 met the inclusion criteria for this review. The nine peripheral biomarkers analyzed for their association with neuronal function include amyloid beta 42 (Aβ42), amyloid beta 40 (Aβ40), Aβ42/Aβ40 ratio, total Tau (t-Tau), phosphorylated tau 181 (p-tau 181), neurofilament light (NfL), brain-derived neurotrophic factor (BDNF), S100B, and soluble receptor for advanced glycation end products (sRAGE). Our findings reveal that peripheral Aβ42, Aβ42/Aβ40 ratio, NfL, and S100B significantly differ between VCI and non-VCI groups, indicating their potential as blood biomarkers for VCI.

## 1 Introduction

Vascular cognitive impairment (VCI) refers to cognitive deficits associated with cerebrovascular diseases, encompassing a broad range of conditions from vascular mild cognitive impairment to vascular dementia (VaD) (1). It is the second most common cause of dementia after Alzheimer’s disease (AD), accounting for at least 20% of all dementia cases (2). Worldwide, more than 57 million people are affected by dementia, a number projected to exceed 150 million by 2050 (3). Early diagnosis of VCI presents significant challenges, increasing the risk of disability and mortality, which places a substantial burden on both families and society (4, 5). The International Vascular Impairment of Cognition Classification Consensus Study (VICCCS) classifies VCI into mild and severe forms. Severe VCI includes post-stroke dementia, subcortical ischemic vascular dementia, multi-infarct dementia, and mixed dementia (where vascular and neurodegenerative lesions coexist) (6). Clinically, VCI is characterized by attention deficits, impaired information processing, difficulties with complex tasks, and disruptions in thinking and behavior (7). In some cases, patients may also experience mood disorders, such as vascular depression (8, 9).

Cerebral small vessel disease (cSVD) is the predominant pathological foundation of VCI (10). cSVD is categorized into six types, with Type I (arteriosclerosis) and Type II (cerebral amyloid angiopathy) being the most prevalent (11, 12). Emerging evidence indicates that the pathological mechanisms of cSVD involve hypoperfusion/hypoxia, blood–brain barrier (BBB) dysfunction, interstitial fluid (ISF)/cerebrospinal fluid (CSF) drainage obstruction, and vascular inflammation (10). On magnetic resonance imaging (MRI), cSVD typically manifests as white matter hyperintensities (WMHs), cerebral microbleeds (CMBs), subcortical infarcts, lacunes, perivascular space enlargement, and brain atrophy (13). WMH, also known as leukoaraiosis (LA), represents the most common neuroimaging feature of cSVD (14, 15). LA corresponds to specific abnormalities in the white matter, often characterized by multifocal or diffuse changes of varying sizes, primarily located around the ventricles (16). Numerous studies have shown that LA is increasingly prevalent in older adults, with approximately 90% of individuals over 60 exhibiting detectable signs (12). LA has been reported to be closely associated with an elevated risk of cognitive dysfunction, motor gait impairment, stroke, dementia, depression, and even mortality (15, 17–19). The Fazekas scale, based on MRI, is widely utilized in the clinical assessment of LA severity (20). Previous studies, such as the Leukoaraiosis and Disability (LADIS) study, have demonstrated that the severity of LA and the presence of diabetes are independent predictors of cognitive decline in initially non-disabled elderly individuals (21). Another LADIS study suggested that physical activity may reduce the risk of cognitive impairment (primarily VaD) in elderly individuals capable of living independently (22). Consequently, LA is intimately linked to cognitive function (23).

The diagnosis of VCI involves clinical evaluation, neuropsychological testing, and neuroimaging (24). The Montreal Cognitive Assessment (MoCA) and the Mini-Mental State Examination (MMSE) are the most commonly employed neuropsychological tools (25). However, these assessments are influenced by the patient’s age and educational background, and the evaluator’s subjectivity can also affect the accuracy of the results. Additionally, neuroimaging relies on high-quality scans and skilled radiologists, and its high cost limits its widespread clinical application. In contrast, blood biomarkers provide benefits such as accessibility, objectivity, minimal invasiveness, and low testing costs (26). As a result, many studies indicate that blood biomarkers are crucial in diagnosing VCI (24, 27). With the advancement of ultrasensitive technologies, such as single-molecule arrays and electrochemiluminescence analysis, concentrations below femtomolar levels can now be detected, enabling highly sensitive measurements of brain-derived proteins at low concentrations (26). Previous studies have shown that patients with VCI who are amyloid-positive experience a more rapid decline in cognitive function across multiple domains compared to amyloid-negative VCI patients, suggesting that VCI may also involve the pathological mechanisms underlying neurodegenerative diseases (28). Moreover, recent research has identified circulating biomarkers that influence neuronal function, including *β*-amyloid 42 (Aβ42), phosphorylated tau 181 (p-tau181), neurofilament light (NfL), and S100B, as being associated with VCI (29–32). Therefore, this paper aims to evaluate the potential of circulating biomarkers in predicting VCI, focusing on neuronal function, through a systematic review and meta-analysis, thereby providing some assistance for the early diagnosis and treatment of VCI.

## 2 Methods

### 2.1 Search strategy

The following keywords were combined for this study: (dementia OR cognitive impairment OR cognitive decline OR cognitive disorder OR cognitive dysfunction OR cognitive deficit) AND (vascular OR strokes OR stroke OR cerebrovascular accident OR brain vascular accident OR apoplexy OR cerebral infarction OR brain infarction OR brain hemorrhage OR cerebral hemorrhage OR hemorrhage) AND (biomarkers OR biomarker OR serum OR plasma OR circulation OR circulating OR peripheral OR whole blood). Four major databases, PubMed, Embase, Web of Science, and Cochrane Library, were searched for all research up until December 31, 2023.

### 2.2 Inclusion and exclusion criteria

The following were the inclusion criteria for the studies in this systematic review: (1) all biomarkers were derived from blood, including whole blood, serum, and plasma; (2) the study type was case–control, cross-sectional, or cohort; (3) the patients had a confirmed diagnosis of VCI; and (4) the samples included both the VCI group and the control group. The following were the exclusion criteria: (1) reviews, meta-analyses, and systematic reviews; (2) case reports and conference abstracts; (3) animal experimentation studies; (4) intervention experiments; (5) papers written in languages other than English; and (6) lack of access to full text or study data.

### 2.3 Data extraction

Data extraction was conducted independently by two researchers (W.H. and L.L.). The collected data comprised the first author’s surname, the publication year, the country of publication, the study type, the gender ratio, the mean age, the sample size, the diagnostic method for VCI, the sample source, and the biomarkers involved. For biomarkers identified in two or more articles, the mean and standard deviation (SD) of concentrations and the sample size for each group were extracted. Standard methods were used to estimate means and SDs when biomarker concentrations were reported in alternative formats, such as median or interquartile range (33, 34). Any conflicts during the process were resolved through continuous discussions involving all authors.

### 2.4 Quality assessment

The quality of the studies included in our review was evaluated using the Newcastle-Ottawa scale. All the studies had scores ranging from 7 to 9, suggesting good quality. This information can be found in Supplementary Table S1. Furthermore, we have previously registered the systematic review program in PROSPERO (registration number CRD42024568815).

### 2.5 Statistical analysis

The data were analyzed using RevMan 5.4 software. To account for variations in the assays used across different studies, the standardized mean difference (SMD) was selected as the effect size for comparing biomarker levels, along with the corresponding 95% confidence intervals (CIs). Heterogeneity among the included studies was assessed and quantified using the Cochrane Q test and I^2^ statistic. A random-effects model was applied when I^2^ exceeded 50%, indicating substantial heterogeneity. Conversely, a fixed-effects model was employed when heterogeneity was deemed insignificant (I^2^ < 50%). Statistical significance was defined as a *p*-value ≤0.05.

## 3 Results

### 3.1 Results of study inclusion

**Figure 1:**
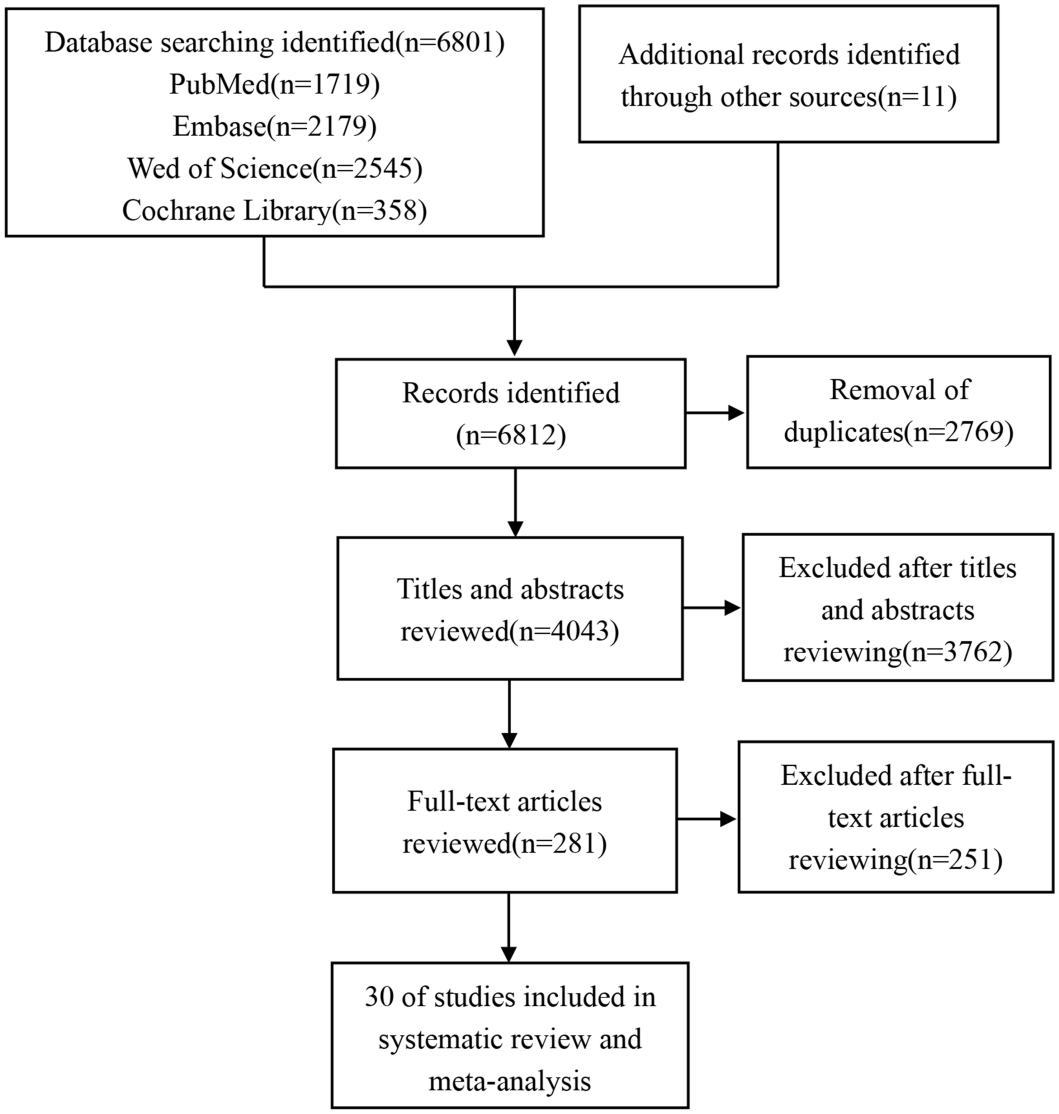
Flow diagram of the study selection.

6,812 studies were identified from various databases: 1,719 from PubMed, 2,179 from Embase, 2,545 from Web of Science, 358 from the Cochrane Library, and 11 from other sources. After removing duplicates, the dataset was reduced to 4,043 studies. After reviewing titles and abstracts, 281 studies were selected for further consideration. Ultimately, 30 articles were included in the final analysis after a comprehensive full-text review (Figure 1).

### 3.2 Characteristics of the included studies reporting potential biomarkers for VCI

Table 1 presents data from research on potential blood biomarkers for VCI. The 30 included articles, published between 2005 and 2023, comprise 10 case–control studies (35–44), 11 cohort studies (29–32, 45–51), and 9 cross-sectional studies (52–60). The studies involved participants from Germany, China, Turkey, Singapore, Italy, and Sweden, with sample sizes ranging from 55 to 5,323. Participants were categorized by gender, and the average age was documented. Each study also specified the type and diagnostic criteria of VCI in patients. Furthermore, the type of blood sample (serum or plasma) and the associated blood biomarkers were recorded.

**Table 1:** Summary of the 30 selected studies reporting potential blood biomarkers for VCI.

Author, Year	Country	Study type	Sample type	Male/female	Mean age	Sample size	Diagnosis of VCI	Specimen	Biomarkers
Emanuele, 2005 (52)	Italy	Cross-sectional study	VaD	136/268	74.4	404	NINDS-AIREN criteria	Plasma	sRAGE
Bibl, 2007 (35)	Germany	Case-control study	VaD	37/35	70.1	72	NINDS-AIREN criteria	Plasma	Aβ42, Aβ40, Aβ38/Aβ40
Uslu, 2012 (57)	Turkey	Cross-sectional study	VaD	27/40	68.4	67	NINDS-AIREN criteria	Serum	Aβ42, IL-6, TNF-α
Liang, 2013 (53)	China	Cross-sectional study	VaD	162/188	69.3	350	NINDS-AIREN criteria	Plasma	sLRP, sRAGE
Gao, 2015 (40)	China	Case-control study	cSVD-CI	212/205	72.5	417	MRI and MoCA	Serum	S100B, ADMA, TC, TG, LDL, HDL
Shi, 2017 (42)	China	Case-control study	VaD	\	52.1	276	NINDS-AIREN criteria	Serum	S100B
Xu, 2016 (58)	China	Cross-sectional study	VaD	62/59	74.5	121	NINDS-AIREN criteria	Plasma	FBG, HbA1c, LDL, sRAGE
Tang, 2017 (56)	China	Cross-sectional study	PSD	111/61	72.1	172	NINDS-AIREN criteria	Plasma	sRAGE, esRAGE
Chi, 2019 (29)	China	Prospective cohort study	PSCI	45/10	61.2	55	MoCA	Plasma	Aβ42, Aβ40, Aβ42/Aβ40, t-Tau
Chen, 2019 (37)	China	Case–control study	PSD	148/86	65.5	234	NINDS-AIREN criteria	Serum	AChE, BChE, AChE activity, BChE activity, ChE activity
Mao, 2020 (48)	China	Prospective cohort study	PSCI	117/71	68.1	188	MoCA	Serum	TC, TG, LDL, HDL, Aβ42, hs-CRP, Hcy, T3, T4, FT3, FT4, TSH,
Zuliani, 2020 (60)	Italy	Cross-sectional study	VaD	371/327	77.0	598	NINDS-AIREN criteria	Serum	BACE1
Ma, 2020 (54)	China	Cross-sectional study	VaD	109/67	69.4	176	NINDS-AIREN and DSM-5 criteria	Serum	NfL, TC, TG, LDL, HDL, FBG
Wang, 2020 (43)	China	Case–control study	VaD	107/65	63.3	172	NINDS-AIREN and ICD-11	Serum	NRG 1, FBG, HDL, LDL
Shao, 2020 (55)	China	Cross-sectional study	VaD	109/79	73.1	188	NINDS-AIREN and DSM-5 criteria	Serum	NPTX2, FT3, FT4, TSH, FBG, HbA1c, HDL, LDL, TG, TC
Holm, 2020 (46)	Sweden	Prospective cohort study	VaD	3731/1592	69.3	5,323	DSM-4 criteria	Plasma	MR-PENK A, NT-PTA
Chua, 2020 (38)	Singapore	Case–control study	VaD	175/209	72.6	384	NINDS-AIREN criteria	Plasma	S1Ps, IL-6, IL-8, TNF
Wang, 2021 (31)	China	Prospective cohort study	PSCI	893/801	64.0	1,694	MoCA	Plasma	NfL, HbA1c, hs-CRP, Hcy
Wang, 2021 (49)	China	Prospective cohort study	PSCI	174/130	64.9	304	TICS-40	Serum	NfL
Zhong, 2021 (51)	China	Prospective cohort study	PSCI	433/184	60.0	617	MoCA, MMSE	Plasma	choline, betaine, TMAO
Zhao, 2021 (59)	China	Cross-sectional study	VaD	126/55	67.6	181	NINDS-AIREN and DSM-5 criteria	Serum	NCAM, FBG, HDL, LDL
Huang, 2022 (30)	China	Prospective cohort study	PSCI	97/39	58.8	136	MoCA	Plasma	Aβ42, Aβ40, Aβ42/Aβ40, t-Tau, p-tau 181, BDNF
Dong, 2022 (45)	China	Prospective cohort study	PSCI	413/180	60.1	593	MoCA	Plasma	NPY
Jiang, 2022 (47)	China	Prospective cohort study	PSCI	157/107	65.0	264	MoCA	Plasma	NfL, HbA1c, hs-CRP, Hcy
Cao, 2022 (36)	China	Case-control study	cSVD-CI	144/125	68.5	269	MRI and MoCA	Serum	Hsp70, Hcy, hs-CRP, HDL, LDL, TG, TC
Liu, 2022 (41)	China	Case-control study	VaD	98/80	68.6	178	DSM-5 criteria	Serum	BDNF, Hcy, NO, IFN-γ
Xu, 2023 (44)	China	Case-control study	PSCI	65/55	76.0	120	MMSE	Serum	TC, TG, LDL, HDL, UA, Cr, FBG, WBC, Hb, Aβ42, p-tau 181
Chua, 2023 (39)	Singapore	case–control study	VaD	240/286	73.7	526	NINDS-AIREN criteria	Plasma	NfL, p-tau 181
Li, 2023 (32)	China	prospective cohort study	PSCI	85/73	72.3	158	MoCA	Serum	FPG, HbA1c, TC, TG, LDL, HDL, Hcy, S100B
You, 2023 (50)	China	prospective cohort study	PSCI	418/182	59.9	600	MoCA	Plasma	sDPP4

Abbreviations: VaD: vascular dementia, NINDS-AIREN: National Institute of Neurological Disorders and Stroke and Association Internationale pour la Recherché et l’Enseignement en Neurosciences, sRAGE: soluble receptor for advanced glycation end products, Aβ: amyloid beta, IL-6: interleukın-6, TNF-α: tumor necrosis factor-alpha, sLRP: Soluble low density lipoprotein receptor-related protein, cSVD-CI: cerebral small vessel disease with cognitive impairment, MRI: magnetic resonance imaging, MoCA: Montreal Cognitive Assessment, ADMA: asymmetric dimethylarginine, TC: Total cholesterol, TG: Triglyceride, LDL: Low-density lipoprotein, HDL: High-density lipoprotein, FBG: fasting blood glucose, HbA1c: glycated hemoglobin, PSD: post-stroke dementia, esRAGE: endogenous soluble receptor for advanced glycation end products, PSCI: post-stroke cognitive impairment, t-Tau: total Tau protein, AChE: Acetylcholinesterase, BChE: Butylcholinesterase, ChE: Cholinesterase, hs-CRP: Hypersensitive C-reactive protein, Hcy: Homocysteine, T3: Triiodothyronine, T4: Thyroxin, FT3: Free triiodothyronine, FT4: Free thyroxin, TSH: Thyrotropin, BACE1:β-secretase enzyme 1, DSM: Diagnostic and Statistical Manual of Mental Disorders, NfL: neurofilament light, ICD: International Classification of Diseases, NRG 1: neuregulin 1, NPTX2: Neuronal Pentraxin 2, MR-PENK A: Midregional Proenkephalin A, NT-PTA: N-terminal Protachykinin A, S1Ps: Sphingosine-1-phosphates, IL-8: interleukın-8, TNF: tumor necrosis factor, TICS-40: the Telephone Interview of Cognitive Status-40, MMSE: Mini-Mental State Examination, TMAO: trimethylamine Noxide, NCAM: neural cell adhesion molecule, p-tau 181: phosphorylated tau 181, BDNF: brain-derived neurotrophic factor, NPY: Neuropeptide Y, Hsp70: heat shock protein 70, NO: nitric oxide, IFN-γ: interferon-γ, UA: uric acid, Cr: creatinine, WBC: white blood cells, Hb: hemoglobin, sDPP4: soluble dipeptidyl peptidase-4.

### 3.3 Meta-analysis results of the potential biomarkers

**Figure 2:**
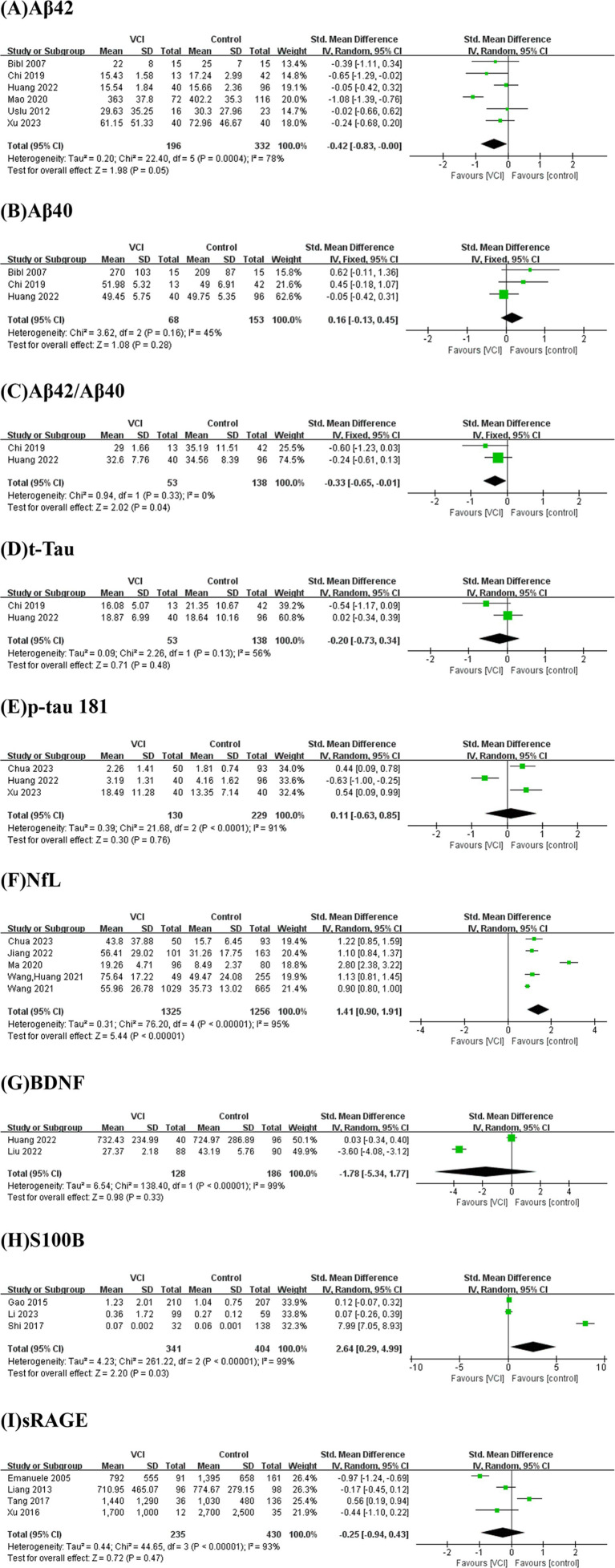
Forest plots for potential biomarkers. Forest plots of the **(A)** Aβ42, **(B)** Aβ40, **(C)** Aβ42/Aβ40, **(D)** t-Tau, **(E)** p-tau 181, **(F)** NfL, **(G)** BDNF, **(H)** S100B, **(I)** sRAGE levels.

Figure 2 presents the results of a meta-analysis conducted on potential blood biomarkers for VCI that were commonly identified across two or more studies. The analysis incorporated nine biomarkers: Aβ42, Aβ40, Aβ42/Aβ40 ratio, total Tau (t-Tau), p-tau 181, NfL, brain-derived neurotrophic factor (BDNF), S100B, and soluble receptor for advanced glycation end products (sRAGE).

#### 3.3.1 Meta-analysis results for Aβ42

**Figure 3:**
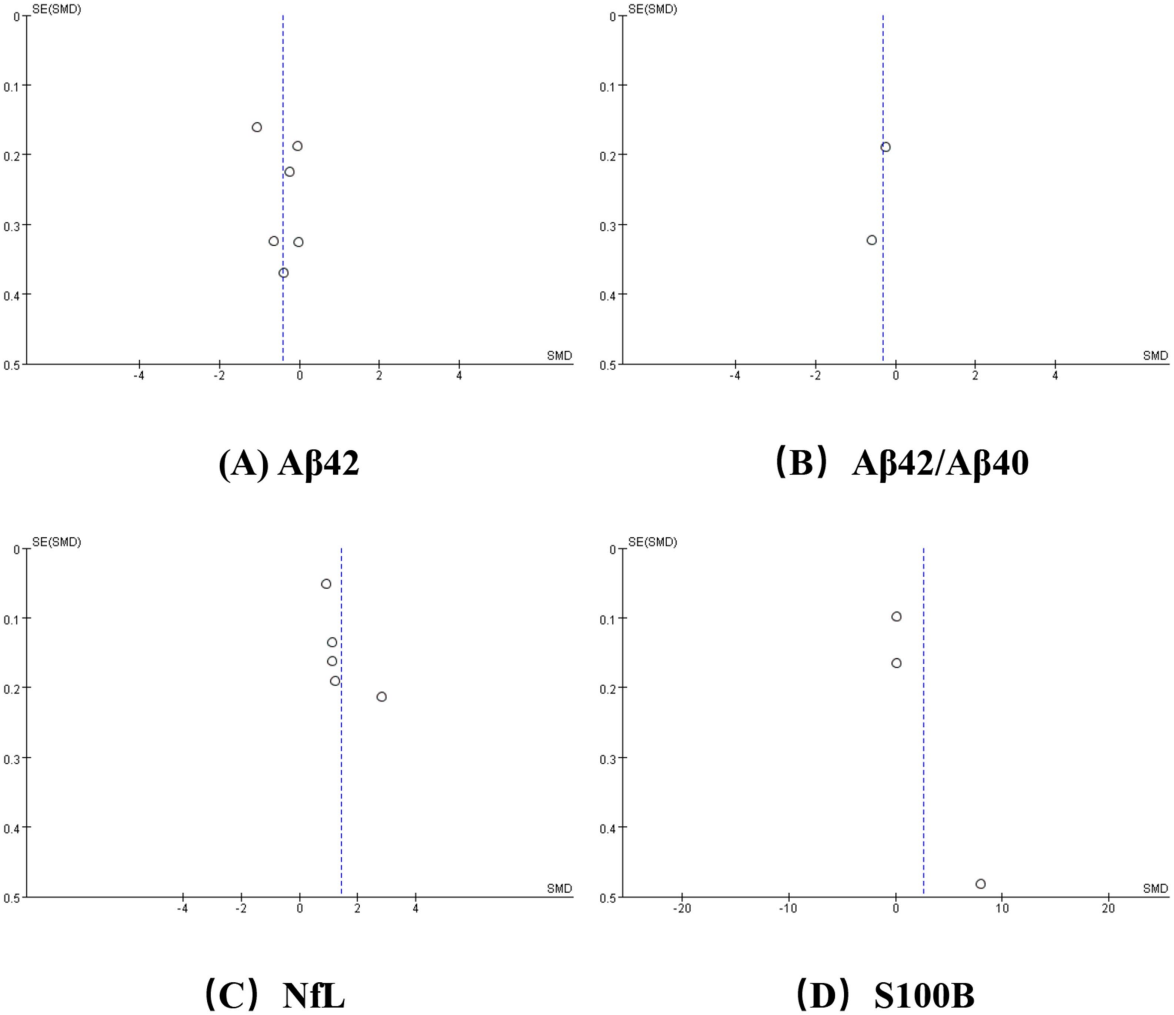
Funnel plot for **(A)** Aβ42, **(B)** Aβ42/Aβ40, **(C)** NfL, **(D)** S100B.

**Figure 4:**
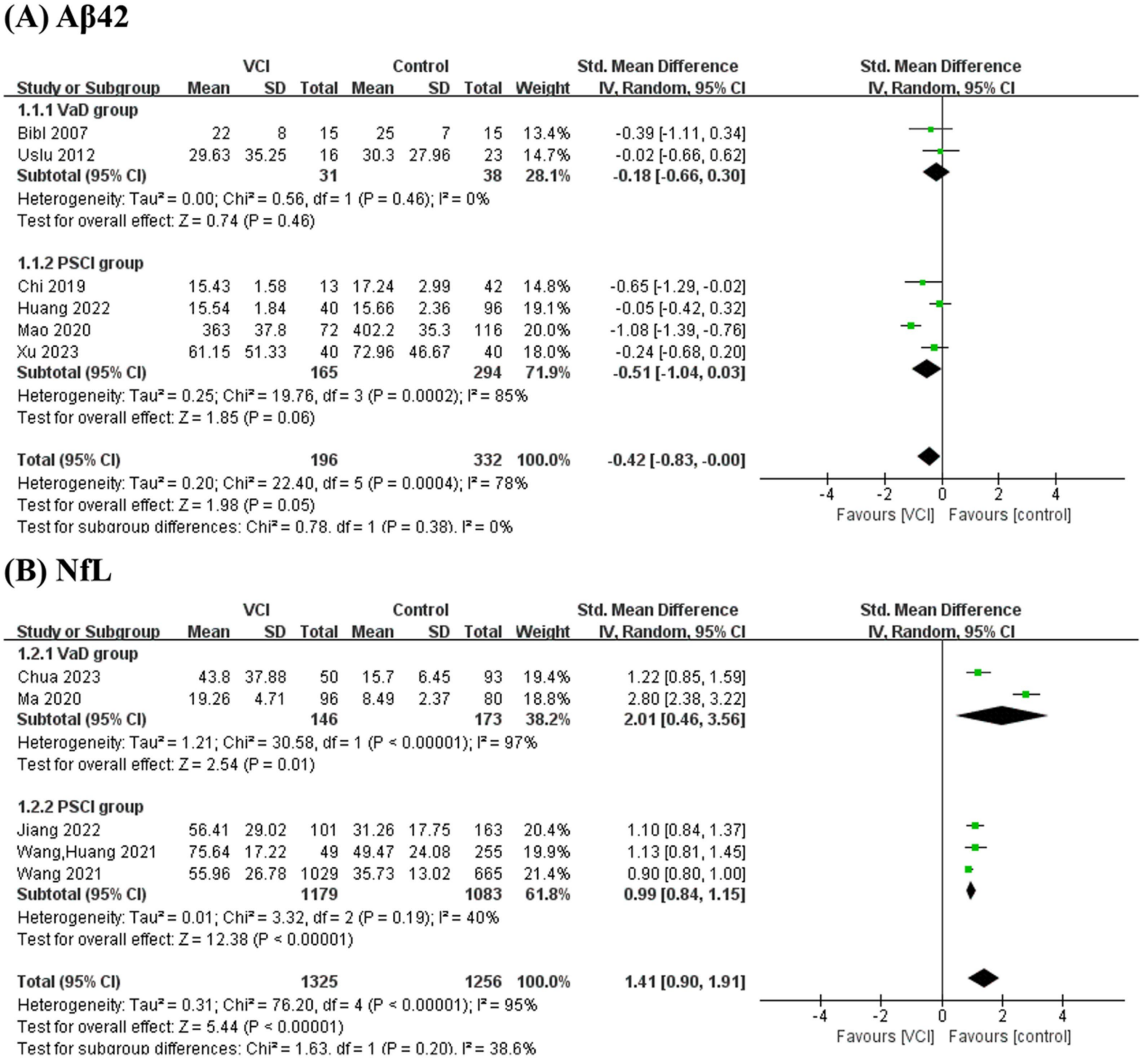
Subgroup analysis for **(A)** Aβ42 and **(B)** NfL.

As depicted in Figure 2A, we performed a meta-analysis of six studies examining peripheral Aβ42 levels involving 196 VCI patients and 332 non-VCI patients. The results demonstrated that Aβ42 levels were significantly lower in the VCI group compared to the control group (SMD = −0.42, 95% CI = (−0.83, 0.00), *p* = 0.05). Figure 3A illustrates that the funnel plot was approximately symmetrical, indicating an absence of significant publication bias. A subgroup analysis based on VCI subtypes revealed no significant differences in peripheral Aβ42 levels between the VaD and post-stroke cognitive impairment (PSCI) groups compared to the control group. The overall difference between subgroups was not statistically significant (*p* = 0.38), as shown in Figure 4A.

#### 3.3.2 Meta-analysis results for Aβ42/Aβ40 ratio

We conducted a meta-analysis of two studies assessing peripheral Aβ42/Aβ40 ratios, including 53 VCI patients and 138 non-VCI patients. As presented in Figure 2C, the results indicated that the Aβ42/Aβ40 ratio was significantly lower in the VCI group relative to the control group (SMD = −0.33, 95% CI = (−0.65,-0.01), *p* = 0.04). The funnel plot, depicted in Figure 3B, suggested no significant publication bias. Given the limited number of studies, further subgroup analysis was not performed.

#### 3.3.3 Meta-analysis results for NfL

We conducted a meta-analysis of five studies examining peripheral NfL levels involving 1,325 VCI patients and 1,256 non-VCI patients. As shown in Figure 2F, the meta-analysis results indicate that NfL levels in the VCI group were significantly higher than those in the control group (SMD = 1.41, 95% CI = (0.90, 1.91), *p* < 0.00001). The funnel plot in Figure 3C displayed poor symmetry, suggesting a certain degree of publication bias. Subgroup analysis based on VCI subtypes revealed that both the VaD group (SMD = 2.01, 95% CI = (0.46, 3.56), *p* = 0.01) and the PSCI group (SMD = 0.99, 95% CI = (0.84, 1.15), *p* < 0.00001) had significantly elevated peripheral NfL levels compared to the control group, while the overall subgroup difference was not statistically significant (*p* = 0.20) (Figure 4B).

#### 3.3.4 Meta-analysis results for S100B

We also conducted a meta-analysis of three studies on peripheral S100B levels involving 341 VCI patients and 404 non-VCI patients. The results showed that S100B levels in the VCI group were significantly higher than those in the control group (SMD = 2.64, 95% CI = (0.29, 4.99), *p* = 0.03) (Figure 2H). Additionally, the funnel plot in Figure 3D indicated no significant publication bias. Due to the limited number of studies, no further subgroup analysis was performed.

#### 3.3.5 Meta-analysis results for Aβ40, t-tau, p-tau181, BDNF, and sRAGE

As illustrated in Figures 2B,D,E,G,I, there were no significant differences between the two groups in terms of Aβ40 (SMD = 0.16, 95% CI = (−0.13, 0.45), *p* = 0.28), t-Tau (SMD = −0.20, 95% CI = (−0.73, 0.34), *p* = 0.48), p-tau181 (SMD = 0.11, 95% CI = (−0.63, 0.85), *p* = 0.76), BDNF (SMD = −1.78, 95% CI = (−5.34, 1.77), *p* = 0.33), and sRAGE (SMD = −0.25, 95% CI = (−0.94, 0.43), *p* = 0.47).

## 4 Discussion

VCI defines the wide spectrum of cognitive disorders caused by different types of cerebrovascular disease and is deemed to be the most common cognitive disorder in the elderly (2). To date, the exact neurochemical basis underlying VCI is not completely clarified. Some of the molecular, biochemical, and electrophysiological abnormalities detected in VCI seem to correlate with disease process and progression. A previous prospective study showed that at least seven different pathologies can predict VCI: large infarcts, lacunar infarcts, microinfarcts, myelin loss, arteriolosclerosis, cerebral amyloid angiopathy (CAA), and perivascular space dilation (61, 62). Growing evidence correlates cerebral hypoperfusion to both cognitive decline and white matter lesions (WMLs) (62). Emerging evidence indicates that various components, such as oxidative stress, neurotransmitter imbalance, neuroinflammation, endothelial dysfunction, and cortical hyperexcitability, play significant roles in VCI (63). Of note, VCI is the only contribution that can be, at least in part, preventable and treatable (64). The search for novel hallmarks of disease process and progression, such as serological, CSF, and instrumental markers, is needed to allow an early, tailored, and accurate screening of VCI patients. Previous meta-analyses have not examined the relationship between VCI and blood biomarkers related to neuronal function. In the present study, we have explored the potential of specific biomarkers associated with neuronal function as indicators. A meta-analysis was conducted on markers examined in two or more of the 30 studies considered.

Cerebral ischemia induces amyloid aggregation, exacerbating inflammatory and neurodegenerative processes in the brain parenchyma, resulting in cognitive dysfunction (65–68). A*β* is a 4 kDa protein produced through the sequential proteolytic cleavage of amyloid precursor protein (APP) by β-secretase and *γ*-secretase (69). Aβ42 and Aβ40 are the two most prevalent isoforms, with Aβ40 being the most abundant (70, 71). Due to its higher hydrophobicity and propensity to aggregate, Aβ42 demonstrates more significant neurotoxicity, with even low concentrations capable of inducing neuronal death (72) and was considered significant potential for the diagnosis of AD (73–77). Huang et al. previously investigated the potential of common AD biomarkers in predicting VCI (30). In this study, we performed a meta-analysis to assess the potential of five AD biomarkers in individually predicting VCI. Our findings indicate that the peripheral levels of Aβ42 and the Aβ42/Aβ40 ratio in the VCI group were significantly lower than those in the control group, while no significant difference was observed in Aβ40 between the two groups. The results of this study suggest that peripheral Aβ42 has potential as a predictor for VCI, aligning with previous findings (29, 35, 44, 48). However, further subgroup analyses did not reveal statistically significant differences between the VaD and PSCI groups compared to the control group. Therefore, the results of this meta-analysis should be interpreted with caution. Moreover, more extensive cohort studies are necessary to validate the ability of Aβ42 to differentiate VCI from AD. It has been reported that the plasma Aβ42/Aβ40 ratio exhibits the strongest correlation with CSF biomarkers (78). Combining Aβ42 with Aβ40 can account for inter-individual differences in Aβ processing and potential pre-analytical confounding factors (79). Previous studies have reported strong consistency between the plasma Aβ42/Aβ40 ratio and amyloid positron emission tomography (PET) status (80). Although the meta-analysis results for the Aβ42/Aβ40 ratio in this study showed significant differences, only two studies were included, both with small sample sizes. Future research should further investigate the relationship between plasma Aβ42/Aβ40 and VCI.

NfL is a crucial component of the neuronal axonal cytoskeleton and is highly concentrated in neuronal axons (81, 82). Pathological processes that result in neuronal axonal damage lead to the release of NfL into the CSF and, at lower concentrations, into the bloodstream (81, 83). Given the strong correlation between NfL levels in the CSF and peripheral blood, coupled with advancements in quantitative detection techniques for plasma NfL (pNfL), research on the role of pNfL in neurodegenerative diseases and brain injuries has been increasing (31, 84–86). Previous studies have identified circulating NfL as a biomarker for AD (73, 85, 87–89). Recently, NfL has also been associated with VCI (31, 39, 47, 49, 54). This study’s meta-analysis reveals that circulating NfL levels are significantly higher in VCI patients than controls. The levels of circulating NfL were found to have a positive correlation with VCI in all of the included studies. Further subgroup analysis indicates that NfL levels in patients with VaD and PSCI are significantly elevated compared to controls, with the SMD for the VaD group being more significant than that for the PSCI group (2.01 vs. 0.99). We hypothesize that the more severe cognitive dysfunction in VaD results in greater neuronal axonal damage, leading to increased NfL release (90). Additionally, a meta-analysis has shown that CSF NfL levels in VaD patients are significantly higher than in AD patients (91). Recently, two studies have reported that plasma NfL levels in VCI patients are significantly elevated compared to those in the AD group, highlighting its potential to distinguish between VCI and AD (39, 92). Meanwhile, a study established a VCI mouse model and found that treatment with the angiotensin 1-7/MAS receptor agonist could reverse cognitive impairment and significantly reduce NfL levels, suggesting that circulating NfL may be a prognostic biomarker for VCI (93). Another longitudinal study showed that baseline NfL levels in cSVD patients could predict changes in MRI biomarkers, cognitive decline, and dementia over a five-year follow-up period; however, no significant changes in NfL levels were observed during the follow-up (94). In conclusion, we believe that NfL is a promising diagnostic and prognostic biomarker for VCI.

S100B is a calcium-binding protein primarily located in astrocytes and Schwann cells, and it is associated with dystrophic axons within Aβ plaques. Upon central nervous system injury, glial cells become activated, and if the BBB is compromised, S100B can be released into the bloodstream (32, 95). This protein has diverse functions, including regulating protein phosphorylation, cell growth, movement, differentiation, the cell cycle, and transcription processes (96). At nanomolar concentrations, S100B functions as a neurotrophic factor; however, higher micromolar concentrations may induce apoptosis (97). Previous studies have demonstrated an association between peripheral S100B levels and VCI (32, 40, 42, 98). Our meta-analysis indicates that serum S100B levels are significantly elevated in the VCI group compared to the control group. Therefore, we propose S100B as a potential biomarker for VCI.

Our meta-analysis found no significant differences between the two groups in t-Tau and p-tau 181. Notably, among the three studies examining peripheral concentrations of p-tau 181, two reported a positive correlation with VCI, while one found the opposite. Thus, future research should continue to investigate this biomarker. Moreover, the meta-analysis did not reveal any statistically significant differences in BDNF and sRAGE between the groups. It is important to note that the number of studies and the overall sample size included in our analysis were limited, and the results may not fully reflect the roles of these biomarkers in VCI. Therefore, larger, multicenter, prospective cohort studies are needed to better understand the genuine relationship between these biomarkers and VCI.

From the studies reviewed here, it appears evident that the biomarkers of neuronal function were deemed to play a role in VCI. Although some of these pathomechanisms are also shared by AD, the findings seem to converge on the possibility that some changes might be specifically involved in VCI patients. Recent studies have identified an association between neuronal functional circulating biomarkers and “asymptomatic” or “covert” cSVD (ccSVD) (99). For instance, one cohort study demonstrated that brain atrophy and WMH are independently associated with plasma NfL levels in cSVD patients with cognitive impairment (100). Additionally, a longitudinal study revealed that plasma NfL levels were significantly elevated in individuals with moderate to severe cSVD burden compared to those without such burden, and these levels positively correlated with CMBs, lacunar infarcts, and moderate to severe WMH (101). Other studies have also suggested that peripheral NfL levels serve as a valuable low-invasive biomarker that can complement MRI findings and may reflect the severity of the cSVD burden (102–104). In addition, a population-based aging study identified a correlation between a lower plasma Aβ42/Aβ40 ratio and higher plasma p-tau217 levels with CAA in individuals exhibiting CMBs (105). Research conducted by Huss et al. revealed that serum levels of glial fibrillary acidic protein (GFAP) in patients with sporadic cSVD were significantly associated with neurocognitive function. This finding suggests that astrocyte dysfunction may play a critical role in the progression of cSVD (106). GFAP, an intermediate filament protein of the astrocytic cytoskeleton, is a specific marker of reactive astrogliosis (107). A prospective cohort study further indicated that serum GFAP is a promising liquid biomarker for sporadic cSVD, as it correlates with clinical severity and cognitive function (108). Consequently, peripheral neuronal functional biomarkers are also crucial in understanding cSVD.

In addition to circulating biomarkers, recent studies have identified neurophysiological and hemodynamic markers as significant predictors of VCI (109). Research suggests that a reduction in cerebral blood flow (CBF) precedes the clinical onset of VCI, indicating that CBF measurement could aid in the early detection of VCI patients (110, 111). The strong correlation between CBF and neuronal function and metabolism underscores its clinical relevance as a marker of brain function (112). Proper regulation of CBF and normal brain metabolism are essential for maintaining cognitive function (112). MRI-based arterial spin labeling (ASL) method is a non-invasive MRI technique that measures tissue perfusion in capillaries and small arteries (109, 113). ASL is a non-invasive MRI technique that measures tissue perfusion in capillaries and small arteries (109, 113). It can be seamlessly integrated into routine brain MRI scans, requiring only 5 min of scanning time (114). ASL offers several advantages, including non-invasiveness, the absence of radiation or tracer use, high reproducibility, and broader accessibility (115). A recent cohort study demonstrated that a decline in ASL-detected CBF is significantly associated with overall cognitive function in VCI patients (116). Several other studies have reported similar findings, suggesting that ASL-based CBF measurement, as a viable alternative to PET (115), holds promise for the early prediction of VCI (111, 117, 118).

Transcranial Doppler ultrasound (TCD), also a non-invasive examination method, may not match the spatial resolution of functional MRI or PET, but it plays a crucial role in hemodynamic assessment due to its excellent temporal resolution (5 milliseconds), ease of operation, and strong resistance to motion artifacts (109). Previous TCD studies suggested that insufficient cerebral perfusion and high vascular resistance may contribute to the development of VCI (119). Furthermore, a meta-analysis of TCD by Fresnais et al. indicated that, compared to cognitively normal elderly individuals, patients with VaD exhibit significantly reduced cerebral blood velocity (CBV) in the MCA and a significantly increased pulsatility index (PI) (120).

At present, there is a deficiency in effective therapies and methods for VCI (121). Recent research has highlighted the significant role of transcranial magnetic stimulation (TMS) in diagnosing and treating VCI (122). TMS is a non-invasive and relatively safe brain stimulation technique that has garnered attention for its ability to selectively induce electrical currents in specific cortical regions through electromagnetic induction (123). Some studies have found that the motor cortex in patients with VaD is more easily excited compared to the control group (resting motor threshold decreased), suggesting a compensatory mechanism in response to ischemic damage and neuronal loss (124). Additionally, research has demonstrated a significant reduction in short-latency afferent inhibition (SAI), an indicator of central cholinergic transmission, in VCI patients (62, 125, 126). Regarding treatment, studies using VaD mouse models have shown that low-frequency repetitive TMS can ameliorate cognitive deficits by upregulating the release of hippocampal BDNF and enhancing the expression of N-methyl-D-aspartate (NMDA) glutamate receptors (122). A recent meta-analysis also indicates that TMS can improve cognitive abilities and daily living activities in stroke patients. The further search for novel hallmarks of disease process and progression, such as serological, instrumental or CSF markers, is needed to allow an early, tailored, and accurate screening of VCI patients. This will also pave the way to innovative the identification of predictors of drug response and therapeutic strategies.

## 5 Limitation

This study acknowledges several limitations. First, most included studies were conducted on Asian populations, which may introduce racial differences that could affect the research outcomes. Second, certain newly identified biomarkers associated with neuronal function, such as *β*-secretase 1 (BACE1), neuropeptide Y (NPY), neural cell adhesion molecule (NCAM), neuregulin 1 (NRG1), neuronal pentraxin 2 (NPTX2), and sphingosine-1-phosphate (S1P), were excluded from the meta-analysis due to the limited number of studies and small total sample size. These findings require further validation in future research. Third, the control groups in these studies predominantly consisted of healthy individuals or stroke patients, leaving the question of whether these biomarkers can distinguish between dementia subtypes unanswered. Fourth, due to the limited number of included articles, a subgroup analysis could not be performed for the Aβ42/Aβ40 ratio and S100B biomarkers. Finally, the predictive capacity of a single biomarker for VCI may be restricted, suggesting that future research might benefit from integrating multiple biomarkers to enhance predictive accuracy.

## 6 Conclusion

In this study, we found that the levels of circulating NfL and S100B in VCI patients were significantly higher than those in non-VCI patients, while the levels of Aβ42 and the Aβ42/Aβ40 ratio were significantly lower in VCI patients compared to non-VCI patients. Therefore, we suggest clinicians focus on these blood biomarkers and integrate neuroimaging and neuropsychological assessments to evaluate the risk of VCI, which may aid in early detection and timely intervention. Additionally, due to the limited number of studies, some other novel blood biomarkers could not be included in the meta-analysis, and we recommend further validation in future research.
