# Supplementary material for: Blood biomarkers for vascular cognitive impairment based on neuronal function: a systematic review and meta-analysis
Source: Front Neurol. 2025 Feb 7;16:1496711. doi: 10.3389/fneur.2025.1496711 (PMC11842260; doi:10.3389/fneur.2025.1496711)
Supplement: Supplementary file 1 [file Data_Sheet_1.PDF]

Supplementary Table S1. Study quality evaluation via the Newcastle-Ottawa Scale

Study [Ref]	Adequate definition of cases	Represent -ativeness of cases	Selection of controls	Defini-tion of controls	Control for important factors	Control for additional factors	Exposure ascertain-ment	Same methods for events ascertain-ment	Non-response rates	Total
Bibl 2007[1]	1	1	0	1	1	0	1	1	1	7
Chi 2019[2]	1	1	1	1	1	1	1	1	1	9
Huang 2022[3]	1	1	1	1	1	1	1	1	0	8
Mao 2020[4]	1	1	1	1	1	1	1	1	1	9
Uslu 2012[5]	1	1	1	1	1	1	1	1	1	9
Xu 2023[6]	1	1	1	1	1	0	1	1	1	8
Jiang 2022[7]	1	1	1	1	1	1	1	1	0	8
Wang[1] 2021[8]	1	1	1	1	1	0	1	1	0	7
Wang[2] 2021[9]	1	1	1	1	1	1	1	1	0	8
Ma 2020[10]	1	1	1	1	1	0	1	1	1	8
Chua 2023[11]	1	1	1	1	1	0	1	1	1	8
Liu 2022[12]	1	1	1	1	1	1	1	1	1	9
Li 2023[13]	1	1	1	1	1	1	1	1	1	9
Shi 2017[14]	1	1	0	1	1	0	1	1	1	7
Gao 2015[15]	1	1	0	1	1	0	1	1	1	7
Xu 2017[16]	1	1	0	1	1	0	1	1	1	7
Tang 2017[17]	1	1	1	1	1	1	1	1	1	9
Liang 2013[18]	1	1	1	1	1	0	1	1	1	8
Emanuele 2005[19]	1	1	1	1	1	0	1	1	1	8
Zuliani1 2020[20]	1	1	1	1	0	0	1	1	1	7
Cao 2022[21]	1	1	1	1	1	1	1	1	1	9
You 2023[22]	1	1	1	1	1	1	1	1	1	9
Dong 2022[23]	1	1	1	1	1	1	1	1	1	9
Zhao 2021[24]	1	1	1	1	1	1	1	1	1	9
Wang 2020[25]	1	1	1	1	1	1	1	1	1	9
Zhong 2021[26]	1	1	1	1	1	1	1	1	1	9
Shao 2020[27]	1	1	0	1	1	1	1	1	1	8
Holm1 2020[28]	1	1	1	1	1	1	1	1	1	9
Chua 2020[29]	1	1	0	1	1	1	1	1	1	8
Chen 2019[30]	1	1	1	1	1	0	1	1	1	8

References

1 Bibl, M. *et al.* Blood-based neurochemical diagnosis of vascular dementia: a pilot study. *Journal of Neurochemistry* **103**, 467-474 (2007). https://doi.org:10.1111/j.1471-4159.2007.04763.x

2 Chi, N.-F. *et al.* Plasma Amyloid Beta and Tau Levels Are Predictors of Post-stroke Cognitive Impairment: A Longitudinal Study. *Frontiers in Neurology* **10** (2019).

https://doi.org:10.3389/fneur.2019.00715
3 Huang, L.-K. *et al.* Plasma Phosphorylated-tau181 Is a Predictor of Post-stroke Cognitive Impairment: A Longitudinal Study. *Frontiers in Aging Neuroscience* **14** (2022). https://doi.org:10.3389/fnagi.2022.889101
4 Mao, L. *et al.* Relationship between β-amyloid protein 1-42, thyroid hormone levels and the risk of cognitive impairment after ischemic stroke. *World Journal of Clinical Cases* **8**, 76-87 (2020). https://doi.org:10.12998/wjcc.v8.i1.76
5 Uslu, S. *et al.* Levels of Amyloid Beta-42, Interleukin-6 and Tumor Necrosis Factor-Alpha in Alzheimer's Disease and Vascular Dementia. *Neurochemical Research* **37**, 1554-1559 (2012). https://doi.org:10.1007/s11064-012-0750-0
6 Xu, M., Yang, L., Zhong, Z. & Ye, M. Research on Diagnostic Markers for Post-Stroke Cognitive Impairment. *Altern Ther Health Med* **29**, 230-235 (2023).
7 Jiang, L. *et al.* Plasma Neurofilament Light Chain Is Associated with Cognitive Impairment after Posterior Circulation Stroke. *Evid Based Complement Alternat Med* **2022**, 2466982 (2022). https://doi.org:10.1155/2022/2466982
8 Wang, Z. *et al.* Plasma Neurofilament Light Chain as a Predictive Biomarker for Post-stroke Cognitive Impairment: A Prospective Cohort Study. *Front Aging Neurosci* **13**, 631738 (2021). https://doi.org:10.3389/fnagi.2021.631738
9 Wang, J. H. *et al.* Circulating Neurofilament Light Predicts Cognitive Decline in Patients With Post-stroke Subjective Cognitive Impairment. *Front Aging Neurosci* **13**, 665981 (2021). https://doi.org:10.3389/fnagi.2021.665981
10 Ma, W. *et al.* Elevated Levels of Serum Neurofilament Light Chain Associated with Cognitive Impairment in Vascular Dementia. *Dis Markers* **2020**, 6612871 (2020). https://doi.org:10.1155/2020/6612871
11 Chua, X. Y. *et al.* Lipidomics profiling reveals distinct patterns of plasma sphingolipid alterations in Alzheimer's disease and vascular dementia. *Alzheimers Res Ther* **15**, 214 (2023). https://doi.org:10.1186/s13195-023-01359-7
12 Liu, J. *et al.* Assessment of Four Serum Biochemical Markers in Elderly Patients with Vascular Dementia after Cerebral Infarction and Their Response to Donepezil and Idebenone. *Journal of Neurological Surgery Part B: Skull Base* **84**, 629-636 (2022). https://doi.org:10.1055/s-0042-1756500
13 Li, Y. *et al.* Correlation Between Cognitive Impairment and Homocysteine and S100B Protein in Patients with Progressive Ischemic Stroke. *Neuropsychiatr Dis Treat* **19**, 209-217 (2023). https://doi.org:10.2147/NDT.S393624
14 Shi, S. *et al.* Expression of S100beta protein in patients with vascular dementia after basal ganglia hemorrhage and its clinical significance. *Exp Ther Med* **13**, 1917-1921 (2017). https://doi.org:10.3892/etm.2017.4207
15 Gao, Q. *et al.* S100B and ADMA in cerebral small vessel disease and cognitive dysfunction. *J Neurol Sci* **354**, 27-32 (2015). https://doi.org:10.1016/j.jns.2015.04.031
16 Xu, X. Y. *et al.* Plasma levels of soluble receptor for advanced glycation end products in Alzheimer's disease. *Int J Neurosci* **127**, 454-458 (2017). https://doi.org:10.1080/00207454.2016.1193861
17 Tang, S. C. *et al.* Elevated Plasma Level of Soluble Form of RAGE in Ischemic Stroke Patients with Dementia. *Neuromolecular Med* **19**, 579-583 (2017).

https://doi.org:10.1007/s12017-017-8471-9

18 Liang, F., Jia, J., Wang, S., Qin, W. & Liu, G. Decreased plasma levels of soluble low density lipoprotein receptor-related protein-1 (sLRP) and the soluble form of the receptor for advanced glycation end products (sRAGE) in the clinical diagnosis of Alzheimer's disease. *J Clin Neurosci* **20**, 357-361 (2013). https://doi.org:10.1016/j.jocn.2012.06.005

19 Emanuele, E. *et al.* Circulating levels of soluble receptor for advanced glycation end products in Alzheimer disease and vascular dementia. *Arch Neurol* **62**, 1734-1736 (2005). https://doi.org:10.1001/archneur.62.11.1734

20 Zuliani, G. *et al.* Increased blood BACE1 activity as a potential common pathogenic factor of vascular dementia and late onset Alzheimer's disease. *Scientific Reports* **10** (2020). https://doi.org:10.1038/s41598-020-72168-3

21 Cao, L. & Sun, Z. Diagnostic Values of Serum Levels of Homocysteine, Heat Shock Protein 70 and High-Sensitivity C-Reactive Protein for Predicting Vascular Cognitive Impairment. *Neuropsychiatric Disease and Treatment* **Volume 18**, 525-533 (2022). https://doi.org:10.2147/ndt.S354022

22 You, S. *et al.* Plasma sDPP4 (Soluble Dipeptidyl Peptidase-4) and Cognitive Impairment After Noncardioembolic Acute Ischemic Stroke. *Stroke* **54**, 113-121 (2023). https://doi.org:10.1161/strokeaha.122.040798

23 Dong, W. *et al.* Plasma neuropeptide Y and cognitive impairment after acute ischemic stroke. *J Affect Disord* **317**, 221-227 (2022). https://doi.org:10.1016/j.jad.2022.08.052

24 Zhao, J., Lu, W., Li, J., Liu, L. & Zhao, X. Decreased Serum NCAM Levels Associated with Cognitive Impairment in Vascular Dementia. *Dis Markers* **2021**, 2792884 (2021). https://doi.org:10.1155/2021/2792884

25 Wang, X. *et al.* Increased Levels of Serum Neuregulin 1 Associated with Cognitive Impairment in Vascular Dementia. *Biomed Res Int* **2020**, 6683747 (2020). https://doi.org:10.1155/2020/6683747

26 Zhong, C. *et al.* Choline Pathway Nutrients and Metabolites and Cognitive Impairment After Acute Ischemic Stroke. *Stroke* **52**, 887-895 (2021). https://doi.org:10.1161/strokeaha.120.031903

27 Shao, K., Shan, S., Ru, W. & Ma, C. Association between serum NPTX2 and cognitive function in patients with vascular dementia. *Brain Behav* **10**, e01779 (2020). https://doi.org:10.1002/brb3.1779

28 Holm, H. *et al.* High circulating levels of midregional proenkephalin A predict vascular dementia: a population-based prospective study. *Scientific Reports* **10** (2020). https://doi.org:10.1038/s41598-020-64998-y

29 Chua, X. Y. *et al.* Immunomodulatory sphingosine-1-phosphates as plasma biomarkers of Alzheimer's disease and vascular cognitive impairment. *Alzheimers Res Ther* **12**, 122 (2020). https://doi.org:10.1186/s13195-020-00694-3

30 Chen, Y. C. *et al.* Serum Level and Activity of Butylcholinesterase: A Biomarker for Post-Stroke Dementia. *J Clin Med* **8** (2019). https://doi.org:10.3390/jcm8111778
